# A quantitative assessment of torque-transducer models for magnetoreception

**DOI:** 10.1098/rsif.2009.0435.focus

**Published:** 2010-01-19

**Authors:** Michael Winklhofer, Joseph L. Kirschvink

**Affiliations:** 1 Department of Earth and Environmental Sciences, Ludwig-Maximilians-University, 80333 Munich, Germany; 2 Division of Geological and Planetary Sciences, California Institute of Technology, Pasadena, CA 91125, USA

**Keywords:** magnetic orientation, biogenic magnetite, mechanosensitive ion channels, cytoskeleton, otolith, radical pairs

## Abstract

Although ferrimagnetic material appears suitable as a basis of magnetic field perception in animals, it is not known by which mechanism magnetic particles may transduce the magnetic field into a nerve signal. Provided that magnetic particles have remanence or anisotropic magnetic susceptibility, an external magnetic field will exert a torque and may physically twist them. Several models of such biological magnetic-torque transducers on the basis of magnetite have been proposed in the literature. We analyse from first principles the conditions under which they are viable. Models based on biogenic single-domain magnetite prove both effective and efficient, irrespective of whether the magnetic structure is coupled to mechanosensitive ion channels or to an indirect transduction pathway that exploits the strayfield produced by the magnetic structure at different field orientations. On the other hand, torque-detector models that are based on magnetic multi-domain particles in the vestibular organs turn out to be ineffective. Also, we provide a generic classification scheme of torque transducers in terms of axial or polar output, within which we discuss the results from behavioural experiments conducted under altered field conditions or with pulsed fields. We find that the common assertion that a magnetoreceptor based on single-domain magnetite could not form the basis for an inclination compass does not always hold.

## 1. Introduction

In the nearly 50 years since Lowenstam (1962) reported the presence of the ferromagnetic mineral magnetite (Fe_3_O_4_) as a hardening agent in the radular teeth of the Polyplacophoran molluscs (the chitons), and suggested it might possibly be used as a magnetic field sensor, it has been found as a matrix-mediated biological precipitate in a plethora of living organisms, including insects (Gould *et al*. 1978), vertebrates (Walcott *et al*. 1979), bacteria (Frankel *et al*. 1979) and even protists (Bazylinski *et al*. 2000). Parallel but totally separate developments in the geophysical field of rock and mineral magnetism during the 1960s and 1970s gradually led to the understanding of the size, shape and chemical properties for magnetite that would produce the uniformly magnetized crystals (termed ‘single-domain’ particles) that were responsible for holding much of the stable, ancient magnetization in rocks (Evans & McElhinny 1969; Butler & Banerjee 1975), providing the basis for determining the deep-time history of the geomagnetic field (the science of palaeomagnetism). It quickly became apparent that natural selection for size, shape, chemistry, crystallographic orientation and several other properties of biological magnetite had converged on the same solutions for producing single-domain crystals in magnetotactic bacteria (Kirschvink & Lowenstam 1979), confirming both the geophysical work and the role of the ferromagnetic materials in their magnetic response.

The typical bacterial geometry is to string the membrane-bound crystals (termed magnetosomes) together into linear chains (Blakemore 1975), supported from magnetostatic collapse by intracellular cytoskeletal filaments (Kobayashi *et al*. 2006; Scheffel *et al*. 2006), allowing their vector magnetic moments to sum linearly, increasing the cellular magnetic moment (Frankel *et al*. 1979). Despite advances in rock magnetic theory and electron microscopy, this basic observation has stood the test of time very well over the past 30 years (Kopp & Kirschvink 2008). Clean-laboratory-based extraction studies, aided by superconducting magnetometry, have identified strings of single-domain magnetite crystals similar to those in the magnetotactic bacteria in the frontal tissues of migratory fish, which are the most free of inorganic contamination (Walker *et al*. 1984, 1988; Kirschvink *et al*. 1985; Mann *et al*. 1988; Diebel *et al*. 2000). In contrast, the densely interacting crystals of biogenic magnetite in chiton teeth were found to be too weakly and randomly magnetized to serve as a compass (Kirschvink & Lowenstam 1979), as are the large, detrital grains of titanomagnetite sometimes found in the vestibular organs of elasmobranch fish (Vilches-Troya *et al*. 1984; Hanson *et al*. 1990).

It is therefore not surprising that various physiological arrangements of single-domain biogenic magnetite have been suggested as a basis for geomagnetic field sensitivity in animals (Gould *et al*. 1978; Kirschvink 1979, 1992; Yorke 1979; Kirschvink & Gould 1981; Edmonds 1992, 1996; Walker *et al*. 2002; Binhi 2006; Walker 2008). Fundamentally, the rotation or translation of a magnetic particle must be able somehow to affect the electrical field across a sensory nerve membrane and thereby influence the production of action potentials that are the common currency of all neural activity. At least two basic approaches have been suggested, one in which the action is caused by magnetic torque of the magnetosomes on other cellular structures (like mechanosensitive transmembrane ion channels; Kirschvink 1992), and an indirect one in which the strong magnetic field surrounding the magnetosome(s) alters magnetochemical reactions (e.g. Kirschvink 1994; Binhi 2006) or interacts with much smaller, superparamagnetic particles (Kirschvink & Gould 1981; Kirschvink *et al*. 1993; Shcherbakov & Winklhofer 1999). In lieu of high-resolution ultrastructural data from the magnetite-containing cells thought to be magnetite-based magnetoreceptors, it is worth exploring quantitatively how biological torque transducers based on biogenic magnetite might function. Our focus is mainly on single-domain magnetite (§3), yet for the sake of completeness we start with a more general treatment of the physics of torque transducers.

## 2. Preliminary considerations

### 2.1. Types of magnetic materials eligible for a torque detector

It is useful to first have a look at the type of magnetic material and domain state needed to realize a torque transducer. In order to experience a torque in an external magnetic field **H**, the intracellular magnetic structure must have (i) a permanent magnetic moment **m**, (ii) an anisotropic magnetic susceptibility **χ**, or (iii) both. In the first case, the magnetic torque is given by2.1a
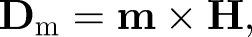

or in scalar notation by2.1b
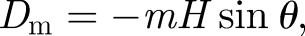

where *H* and *m* are the magnitudes of the vectors **H** and **m**, respectively, and *θ* is the angle between **H** and **m**, reckoned from **H**. The minus sign in equation (2.1*b*) expresses the tendency of the torque to reduce the angle *θ* by turning **m** into alignment with **H**. The single most important example for a structure with permanent magnetic moment and negligible magnetic susceptibility is a chain of single-domain particles of magnetite. The chain need not be a single-stranded magnetosome chain as in magnetotactic spirilla and vibrios, but may have two or even three coherently polarized strands running parallel to each other, as often seen in magnetic cocci and rod-shaped cells (e.g. Hanzlik *et al*. 2002). Magnetite crystals with grain sizes of about 50 nm have stable single-domain behaviour. The upper critical size depends on the aspect ratio and shape (Newell & Merrill 1999; Witt *et al*. 2005) and—in the case of a chain—on the gap width between adjacent crystals (Muxworthy & Williams 2006). Magnetite crystals smaller than about 50 nm (cubes) to 20 nm (elongated; Winklhofer *et al*. 1997) cannot act as stable single domains and show superparamagnetic behaviour, unless stabilized in a linear chain, which helps to extend the lower end of the stability range down to 10–15 nm grain size (Newell 2009). Superparamagnetic crystals cannot retain a stable magnetic moment, but when forming a collective, they have a magnetic susceptibility much larger than a paramagnetic system. Provided that such a collective has shape anisotropy, it qualifies for the type-II torque detector, for which the torque is given by2.2
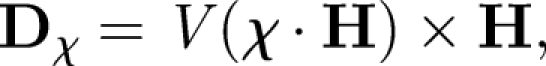

where **χ** is the tensor of the apparent magnetic susceptibility of the particle collective and *V* is its volume (see appendix A for definitions of apparent and intrinsic magnetic susceptibilities). Since **H** × **H** = **0**, we immediately see from equation (2.2) that **χ** needs to be anisotropic to produce a finite torque **D**_**χ**_. This can be achieved by distributing magnetic material of given intrinsic susceptibility over a structure that has a length-to-width ratio different from unity, in which case the apparent magnetic susceptibility is largest along the long axis and smallest along the short axis. A good example here is the dendrites in the upper-beak skin of homing pigeons, which contain numerous clusters of superparamagnetic particles arranged along the axis of the dendrite (Davila *et al*. 2003, 2005; Fleissner *et al*. 2003).

Of course, it is also possible to have a hybrid torque detector, based on both remanent and induced magnetization. Theoretically, this may apply to magnetite particles with grain sizes of about 1 µm and larger, which host magnetic multi-domain (MD) structures. MD particles have been found in the saccular otolith mass among proper calcitic otoliths of elasmobranch fish (guitarfish *Rhinobatos* sp., Vilches-Troya *et al*. 1984; dogfish *Squalus acanthias*, Hanson *et al*. 1990). The relatively high titanium content of the MD particles implies that they are of igneous origin (exogenous) rather than biomineralization products. In the squaliform shark, the (titano)magnetite appears to be randomly dispersed in the otolith mass (Hanson *et al*. 1990). In contrast, the titanomagnetite in the ray is concentrated in conspicuous bands along the saccular membrane (Vilches-Troya *et al*. 1984). Iron-rich otoliths have been detected also in the lagenar otolith membrane of birds and teleost fish (Harada *et al*. 2001), but no information was given as to the grain-size spectrum or mineralogical composition. All these authors discussed a possible involvement of the magnetite otoliths in the magnetic sense, but only Hanson *et al*. (1990) provided quantitative estimates, from which they concluded that the magnetic torque in the dogfish otolithic mass is two orders of magnitude lower than the detection threshold. Their calculation was based on the assumption that the magnetic torque would come from the remanent magnetic moment **m** of the magnetite particles (i.e. according to equation (2.1*a*)), which is relatively small for MD particles with randomly aligned magnetic moments (Hanson *et al*. 1990). Besides, an MD particle has a low magnetic moment in relation to its saturation moment anyway.

Below we estimate the torque according to equation (2.2), given that the magnetite-loaded otolithic mass represents a structure that has an anisotropic susceptibility. The magnetic material is assumed to be roughly homogeneously dispersed over the otolith mass with a volume concentration *c* between 1 and 5 per cent. The magnetic layer of the otolith mass is approximated as a general ellipsoid with half-axes 5 mm and 2.5 mm in the surface and 0.1 mm in the depth. The low-field susceptibility *χ* of isotropic MD magnetite is about 3/4*π* G Oe^−1^ (cgs) or 3 (SI) (Heider *et al*. 1996), so that in an Earth-strength magnetic field of *H* = 40 A m^−1^ (SI) or *H* = 0.5 Oe (cgs), the induced magnetization is 120 A m^−1^ (SI) or 0.12 G (cgs), which is 2.5 × 10^−4^ in terms of the saturation magnetization of magnetite (480 G). From the formulae given in appendix A, the magnitude of the in-plane force couple is obtained as approximately 10^3^*kT* for *c* = 1 per cent and as 2 × 10^4^*kT* for *c* = 5 per cent, where *kT* is the thermal energy at body temperature. The out-of-plane force couple is at least an order of magnitude larger than the in-plane couple since the thickness of the magnetic layer was assumed to be much smaller than its in-plane dimensions. However, since hair-cell bundles underlying the otolithic membrane can be stimulated most efficiently by a force applied parallel to the plane of the membrane, it suffices to consider only the in-plane couple, produced by the difference in apparent susceptibility along the short and intermediate axis. Our values of 10^3^*kT* and 2 × 10^4^*kT* for *c* = 1 per cent and *c* = 5 per cent, respectively, appear large compared with the magnetic torque that acts on a magnetotactic micro-organism—typically between 5*kT* and 10*kT* in the geomagnetic field (Frankel & Blakemore 1980). However, one needs to bear in mind that the otolithic membrane is a macroscopic structure and not a micrometre-sized object! It turns out that our values are actually dwarfed by the value of 10^7^*kT*, which Hanson *et al*. (1990) obtained according to equation (2.1*a*) on the basis of the magnetic remanence and from which they arrived at their negative result. Note that although the induced magnetic moment in our case is approximately 10^8^*kT*/Oe (*c* = 1%) and 8 × 10^8^*kT*/Oe (*c* = 5%), the in-plane anisotropy of *χ* is obviously not pronounced enough to produce a large magnetic torque. These considerations show that a magnetite-loaded otolith layer is hardly suitable as magnetomechanical transducer, unless the organisms were able to realign the exogeneous magnetic particles so that their magnetic remanence vectors added up consistently to produce a large remanent moment. Therefore, it is much more likely that exogenous (titano)magnetite helps in the betterment of the sensitivity to gravity and acceleration of the otolith organ by simply increasing the density (Vilches-Troya *et al*. 1984).

It is interesting to compare these mechanosensitive organs in the vertebrates' vestibular sense with Johnston's organ in the antennae of insects, which serves similar purposes, but until recently was assumed to lack statoliths (i.e. dense minerals). Ultrafine-grained magnetic ore minerals and (non-magnetic) silicates, most probably of exogenous origin, have now been found to be associated with Johnston's organ in the migratory ant *Pachycondyla marginata* (Oliveira *et al*. 2009). As discussed earlier, the dense minerals may help to improve the sensitivity of gravireception. Whether or not that organ has the potential to act as a magnetosensitive unit depends on the concentration and type of magnetic material. If that is sufficient to produce a magnetic torque of 10^2^*kT*, it can also produce a physiologically meaningful signal because it acts on structure of dimensions 10 µm.

### 2.2. ‘Polar’ and ‘axial’ response

Next, it is important to discuss the qualitative difference between the magnetic torque acting on a permanent magnet (equation (2.1*a*)) and the one acting on a structure with anisotropic magnetic susceptibility (equation (2.2)). As we shall see, this difference can explain the two different types of magnetic compass orientation behaviour in animals. The biological compass of arthropods (spiny lobster, Lohmann *et al*. 1995; honey bees, Kirschvink & Kobayashi-Kirschvink 1991), teleost fish (salmon, Quinn *et al*. 1981) and even mammals (mole rat, Marhold *et al*. 1997; bats, Wang *et al*. 2007) was shown to be sensitive to the polarity of the magnetic field. The so-called inclination compass, first discovered in migratory birds (Wiltschko & Wiltschko 1972), however, is blind to the polarity of the magnetic field and only captures the axial orientation of field lines in space. The inclination compass has been reported also in sea turtles (Light *et al*. 1993) and Amphibia (newts, Phillips 1986; urodeles, Schlegel 2008). Interestingly, normal inclination compass-based orientation in birds is absent when these are tested under total darkness and is supplanted by a so-called fixed-direction response (Stapput *et al*. 2008), which—contrary to what the name suggests—shifts when the ambient magnetic field is shifted. Importantly, the ‘fixed-direction’ response is sensitive to field polarity (Stapput *et al*. 2008).

In order for a magnetoreceptor mechanism to be consistent with the inclination compass, it must satisfy the functional relations2.3a
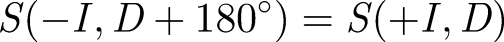

and2.3b
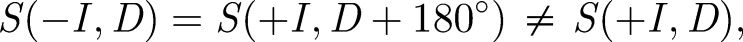

where *S* is the ‘signal’ produced by the mechanism under a given field condition, *I* is the inclination of the field lines with respect to the horizontal and *D* is the declination angle of magnetic north from geographical north. Condition (2.3*a*) expresses the axial nature of the inclination compass, i.e. it is blind to the magnetic polarity of the field lines. Condition (2.3*b*) states that the signal produced in a magnetic field with its vertical component reversed is indistinguishable from the one produced in a magnetic field with horizontal component reversed, but different to the one produced under normal field conditions.

Using trigonometric identities (see appendix B), it can be easily shown that *V*(*χ*·**H**) × **H** for a torque detector of type II satisfies conditions (2.3*a*,*b*). Thus, a torque mechanism on the basis of induced magnetization (equation (2.2)) is consistent with the inclination compass, but at odds with a polarity-sensitive compass. This was suggested for the superparamagnetic torque-transducer model presented in Davila *et al*. (2005). It should be mentioned that magnetoelastic deformation of an assemblage of superparamagnetic particles would also be in accord with the inclination compass (Kirschvink & Gould 1981; Shcherbakov & Winklhofer 1999), since elastic strain is an axial property by default.

A polarity-sensitive biological compass based on magnetic particles requires these to have a remanence, which acts as a bias. However, in order to transmit the bias, the transduction mechanism has to be sensitive to the rotational sense of the torque vector (or equivalently, to the sign of the force couple produced by the torque vector), in which case *S* = **m** × **H**, which in turn is at odds with an inclination compass (see appendix B). However, if only the magnitude of the torque is transduced, i.e. if *S* = |**m** × **H**|, then a remanence-based system satisfies conditions (2.3*a*,*b*) and is in accord with the inclination compass. Thus, remanence is necessary, but not yet sufficient for a polarity-sensitive compass. While our considerations apply to the level of transduction, we emphasize that it may also depend on the measurement principle as to whether or not a remanence-based torque detector is used for a polar or an axial compass. For example, Edmonds (1992) has shown that an axial compass can be realized with a remanence-based ‘null detector’. The measurement principle he suggests is such that the bird looks for the position at which the remanence vector of a given receptor cell is parallel or antiparallel to the external field, to make **m** × **H** = 0.

**Table 1.** Expected compass responses for a torque detector based on magnetic remanence and anisotropic susceptibility, respectively. Realization of a polar compass requires a remanence-based detector that transduces both magnitude and rotational sense of the torque, *m* × *H* (equation (2.1*a*)). Without transducing the sign (i.e. the rotational sense) of the torque, a remanence-based detector produces axial output information only. |*m* × *H*|, magnitude of torque and |*m* × *H*| = 0, null-detector principle according to Edmonds (1992).

	remanence, *m*			anisotropic susceptibility *χ*
	*m* × *H*	|*m* × *H*|	|*m* × *H*| = 0	*χH* × *H*
polar compass	+	−	−	−
axial compass	−	+	+	+

The important conclusion from this section is that the observation of an inclination compass puts no tight constraints on the underlying magnetoreception principle (see table 1 for a summary). Besides, a radical-pair mechanism is also consistent with an axial compass, as was first pointed out by Schulten *et al*. (1978). The only candidate structures of a magnetoreceptor that can be ruled out as basis of the inclination compass are the magnetite-loaded dendrites in the upper-beak skin of birds, as was recently demonstrated by Zapka *et al*. (2009). They severed the ophthalmic branch of the trigeminal nerve to inhibit transmission of signals from the magnetite-loaded dendrites to the brain and found the inclination compass to be unaffected.

### 2.3. Effect of a magnetic pulse

Behavioural studies on impulse-magnetized animals have clearly shown that magnetic material is involved in magnetic orientation behaviour (Walcott *et al*. 1988; Kirschvink & Kobayashi-Kirschvink 1991). After treatment with a brief but strong magnetic pulse, migratory birds and homing pigeons (Wiltschko *et al*. 1994, 2002, 2007, 2009; Beason *et al*. 1997; Munro *et al*. 1997*a*), sea turtles (Irwin & Lohmann 2005) and bats (Holland *et al*. 2008) showed headings significantly different from controls and the pulse effect lasted at least a few days. As in §2.2, we can now ask how a torque mechanism is affected by a pulse. Let us assume for the sake of simplicity that magnetic remanence is carried by a magnetosome chain. The polarity of a magnetosome chain will be switched if the angle between the magnetic moment and the pulse field is greater than 90°, but remains unaffected if the pulse is applied at smaller angles. Independent of the field angle, the magnitude of the magnetic moment is unaffected in a well-organized magnetosome chain, in which all crystals have the same polarity before pulsing. Thus, the only parameter that may change upon pulsing is the polarity of the chain so that the overall effect again depends on whether the transduction mechanism is sensitive to the rotational sense or just to the magnitude of the torque. If it is only the magnitude of the torque that is transduced, then a pulse has no effect (except during the short exposure time). We note that the orientation behaviour of young, inexperienced birds indeed remained unaffected by a pulse (Munro *et al*. 1997*b*). Since they solely rely on the inclination compass, one can safely conclude that the inclination compass is not affected by a pulse. Hence, an axial torque detector is consistent with an inclination compass as well as with the absence of a pulse effect in young birds. However, the absence of a pulse effect does not make a case for an axial torque detector because a radical-pair compass is consistent with these observations, too. The situation in sea turtles (*Caretta caretta*) appears different. Despite their axial compass response under dark conditions (Light *et al*. 1993), they were disoriented after multiple pulsing in two orthogonal directions when observed under dark conditions (Irwin & Lohmann 2005). This observation is consistent with an axial torque detector based on a group of superparamagnetic clusters, which can be disrupted by a pulse (Davila *et al*. 2005; see also discussion in Winklhofer 2009).

If, on the other hand, the transduction mechanism is sensitive to polarity, then those receptor cells that have a switched magnet will produce wrong signals, while those that have not been affected continue to give correct signals. Assuming that the animal does not know which cells produce correct or spurious results, we can expect it to recalibrate the output signals, which may well take a few days. It would be interesting to have pulsed animals ‘regenerate’ under altered field conditions so as to find out whether they recalibrate their magnetic orientation sense to the altered field conditions or whether they can restore the original calibration. Either way, a polarity-sensitive torque transducer is consistent with the presence of a transient pulse effect, provided that the pulse does no damage to the sensory cells, in which case also an animal equipped with an axial torque detector would be affected by a pulse. Since the pulse affects only experienced birds, whose ‘magnetic map’ relies on non-compass information as well, a polarity-sensitive torque transducer is consistent with a ‘magnetic map’, too.

## 3. Torque-transduction principles

The magnetic torque produced in the receptor cell needs to be transduced into a receptor potential and several ideas have been proposed of how a torque mechanism may be connected to a transduction pathway. These may be subsumed under the two categories ‘mechanosensory’ and ‘orientation sensitive’. Models of the first category assume that the magnet is coupled to a mechanosensory transduction pathway that transduces the mechanical force (or stress) caused by the torque. This principle was exploited in the earliest man-made magnetometers, which used the twist angle of a torsion spring (e.g. a slender quartz rod) attached to a magnet as a direct measure of the magnetic torque. The majority of published transducer models belong to this category. Rather than to discuss them chronologically, it is more instructive to first theoretically analyse the generic behaviour of the elementary magnetoelastic torque balance (§3.1). By way of example, we then focus on two specific models based on mechanically gated ion channels (Kirschvink 1992; see §3.3.2).

The second category of models assumes that the intracellular magnet can rotate relatively easily so that it can be aligned with the ambient field. The transduction of the magnetic field is then assumed to occur by secondary processes that depend sensitively on the orientation of the magnet. These secondary processes may be magnetically sensitive chemical reactions involving paramagnetic radicals, as proposed by Binhi (2006). To achieve maximum angular sensitivity, Binhi's model for the compass requires that the intracellular magnet be not completely free to rotate, but be coupled to a soft elastic matrix. We will discuss Binhi's model in §3.3.1 after having provided a general analysis of the magnetoelastic equations, which form the basis of all torque-transducer models.

### 3.1. Generic equations

For a permanent magnet coupled to elastic material, the magnetic torque in its simplest form can be written as3.1
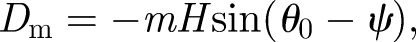

where *ψ* is the deflection of **m** out of its resting orientation, defined as the orientation for which the elastic energy is minimum (*ψ* = 0), and *θ*_0_ is the angle between **H** and the resting orientation of **m**, reckoned in the same rotational sense as *ψ*. For a susceptibility-based system in its simplest form, we have *D*_m_ = −(1/2)*ΔχH*^2^*V* sin 2(*θ*_0_ − *ψ*), where *Δχ* is the difference in apparent susceptibility along the long and short axis, respectively. Analytical expressions of magnetoelastic equilibrium solutions for susceptibility-based systems have been already provided in Shcherbakov & Winklhofer (2004), which is why we here exclusively concentrate on torque mechanisms based on permanent magnets.

The elastic energy of the simplest torque receptor is of the generic form3.2
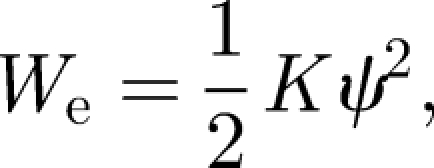

from which the restoring torque is obtained as3.3
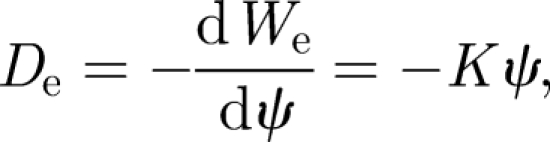

where the generic spring constant *K* may be the torsional stiffness or the bending (flexural) stiffness of the elastic element, depending on the geometrical constraints imposed on it, which in turn depend on how the elastic element is attached to the magnet at its free end (while the other end is fixed and acts as an elastic pivot). As we show in §3.3.2, the elastic parameters of the transducing elements can be easily absorbed into *K* so that equation (3.3) contains all torques related to elastic processes.

In the classical torsion magnetometer, the magnet is constrained to rotate in the plane normal to the long axis **z** of the torsion spring by attaching it to the free end of the spring with its dipole axis perpendicular to **z**. This implies that the torsion magnetometer is sensitive only to the field component perpendicular to the long axis **z**, but not to the full vector. The spring constant *K* of the torsion spring is given by 2*GI/L*, where *G* is its shear modulus, *L* its length and *I* the second moment of inertia of its cross section, which, for a circular cross section of radius *r*, is given by (*π*/4)*r*^4^. If, however, the magnet is fixed at the free end of the elastic element such that its dipole axis is collinear with **z**, it has two degrees of rotational freedom and so is sensitive to the full field vector. The elastic element responds by bending, and so its spring constant *K* is given by *EI/L*, where *E* is Young's modulus. The quantity *EI* is commonly referred to as flexural rigidity. For cytoskeletal filaments, *E* is typically 2 GPa (actin, tubulin), while the flexural rigidity *EI* is 6 × 10^5^ pN nm^2^ for actin (*r* = 3.5 nm) and 2.6 × 10^7^ pN nm^2^ for tubulin (*r* = 10 nm; e.g. Howard 2001, pp. 31, 121). Owing to the quadratic dependence of *K* on the cross-section area and the inverse dependence on the length, values of biological realizable spring constants *K* can vary over many orders of magnitude. For the magnetic torque detector, we require that *mH* be not dwarfed by *K* because the deformation *ψ* will be of the order of *mH*/*K* (for *mH* ≤ *K*). The solution *ψ*(*θ*_0_; *mH*/*K*) can obtained from balancing the magnetic and elastic torques, *D*_m_ = *D*_e_. An algebraic expression for *ψ*(*θ*_0_; *mH*/*K*) does not exist, but these expressions provide useful approximations,3.4a
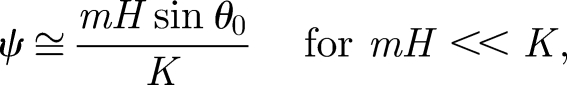

3.4b
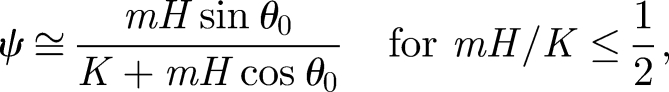

3.4c
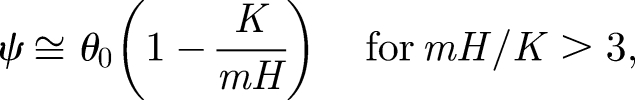

so that a small change **δ*H* in the external field produces a change in deflection by3.5
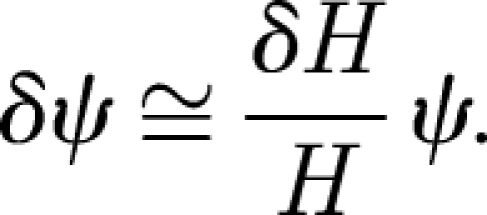



Although expression (3.4*c*) is reminiscent of the asymptotic behaviour of the Langevin function, *L*(*x*) = coth(*x*) − 1/*x*, with *x* = *mH/K*, the Langevin function does not represent a general solution of the problem. For the range 1/2 < *mH/K* < 3, the equilibrium deformation *ψ* has to be found numerically. So far, we have considered the mechanical equilibrium values of *ψ*, but when the energies involved are of the order of 10*kT* or less, it is mandatory that thermodynamic equilibrium values and fluctuation amplitudes be determined as well (see appendix C for technical details). The necessity of the thermodynamic approach was demonstrated in Shcherbakov & Winklhofer (1999), who found that a deformation mechanism on the basis of an individual micrometre-sized superparamagnetic cluster in homing pigeons (Hanzlik *et al*. 2000; Winklhofer *et al*. 2001) would work well from the point of view of equilibrium mechanics, but not so well from the point of view of thermodynamics, unless stabilized by a very high intrinsic magnetic susceptibility or a single-domain particle.

### 3.2. Results: thermodynamic equilibrium deflection

**Figure 1.**
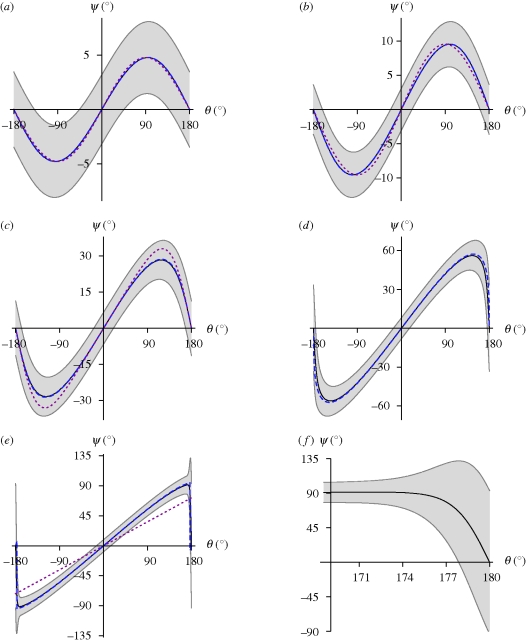
Equilibrium deflection 〈*ψ*〉 of an elastically coupled permanent magnet as a function of the orientation *θ*_0_ of the external field with respect to the rest orientation of the magnet *ψ* = 0, for various values of *mH* and *K*. Ratio *mH/K* increases from (*a*) to (*e*). Solid line, 〈*ψ*〉, thermal equilibrium value of deflection (equation (C 2)); grey area, angular scatter ±*Δψ* about 〈*ψ*〉 (equation (C 4)); blue dashed line, mechanical equilibrium value of deflection *ψ*; red dotted line, approximate mechanical equilibrium deflection according to equation (3.4*a*) in (*a*,*b*) and equation (3.4*b*) in (*c*). Note the strong increase in angular scatter on going towards the antiparallel field orientation (*θ*_0_ = 180°) once *mH/K* ∼1, in (*d*) and particularly in (*e*). (*f*) Zoom-in on the unstable region in (*e*). (*a*) *mH* = 25*kT*, *K* = 300*kT*; (*b*) *mH* = 50*kT*, *K* = 300*kT*; (*c*) *mH* = 25*kT*, *K* = 50*kT*; (*d*) *mH* = 25*kT*, *K* = 25*kT*; (*e*) *mH* = 25*kT*, *K* = 15*kT*.

Figure 1 shows the mechanical and thermodynamic equilibrium values of the magnet's deflection *ψ*(*θ*_0_; *mH*/*K*) as a function of the external field angle *θ*_0_ with respect to the resting position for a range of *K/mH* values and thermal energies. We can see that the mechanical equilibrium values of *ψ* (dashed blue lines in figure 1) are, by and large, identical with the thermodynamic ones. We also find that expressions (3.4*a*–*c*), where applicable, provide decent approximations (dotted red lines in figure 1).

**Figure 2.**
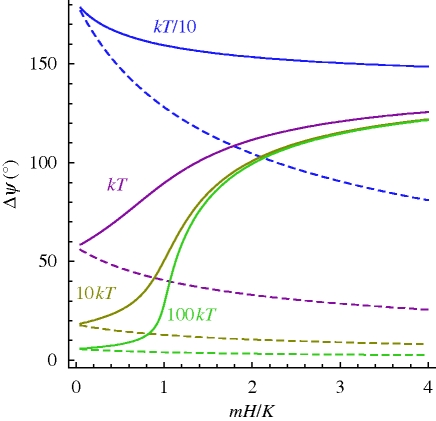
Amplitude of the angular scatter produced by thermal fluctuations as a function of the ratio of magnetic energy *mH* to elastic rigidity *K* for various values of *K*. Solid lines, *Δψ*(*θ*_0_ = 180°), i.e. angular scatter about the equilibrium position when the field is antiparallel to the rest orientation of the magnet, calculated according to equation (D 3*b*). Dashed lines, angular scatter *Δψ*(*θ*_0_ = 0) for the parallel orientation (equation (D 3*a*)).

Importantly, the *ψ*(*θ*_0_) curves are ambiguous for *mH/K* ≤ 1 in the sense that we can always find a ‘virtual’ field orientation 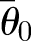 that produces the same deflection of the magnet as the actual field orientation *θ*_0_ does. For *mH/K* ≪ 1, 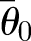 is simply the supplementary angle to *θ*_0_, i.e. 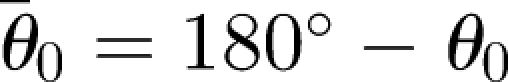. This follows immediately from equation (3.4*a*) because sin *θ*_0_ = sin(180° − *θ*_0_). With larger values of *mH/K* (but still less than 1), *ψ*(*θ*_0_) becomes more asymmetric but still retains its ambiguity. As *mH/K* exceeds 1.5, *ψ*(*θ*_0_) increases monotonically up to *θ*_0_ = 180° − *η* (*η* is small) to plummet to zero as *θ*_0_ approaches 180°. While the ambiguity in *ψ*(*θ*_0_) now is practically confined to the *θ*_0_ = 0° and 180° orientations, both are by no means equivalent, because the parallel one (*θ*_0_ = 0) represents a lower energy state than the antiparallel one (*θ*_0_ = 180°). Therefore, the antiparallel state can be more easily perturbed by thermal fluctuations than the parallel state can. This can be seen in figure 1 from the amplitude *Δψ*(*θ*_0_) of the ‘error margins’ (grey area): *Δψ*(*θ*_0_ = 180°) is always larger than *Δψ*(*θ*_0_ = 0), particularly so for *mH/K* >1, in which case the fluctuation amplitude *Δψ*(*π*) exceeds the amplitude of the maximum signal. Figure 2 shows the behaviour of *Δψ*(*θ*_0_ = 0) and *Δψ*(*θ*_0_ = *π*) as a function of *mH/K* for a range of *K/kT* ratios (see appendix D for an analytical derivation of fluctuation amplitudes). In the quasi-non-thermal regime (see green line in figure 2 for *K* =100*kT*), the angular scatter *Δψ*(*θ*_0_ = 180°) soars as *mH* approaches *K*. With increasing influence of thermal fluctuations, the transition is less sharp (see olive and magenta curves in figure 2 for *K* = 10*kT* and *K* = 1*kT*, respectively) because the system has a large scatter already in the non-magnetic limit *mH/K* = 0, where the angular fluctuations are due solely to thermally induced bending of the elastic element, 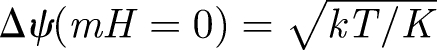. Note that *Δψ*^2^ = *kT/K* = *L/L*_p_, where *L*_p_ = *EI/kT* is the so-called thermal persistence length, which defines the characteristic length scale related to thermal bending of a (non-magnetic) filament. For example, *Δψ* amounts to 0.14 rad (8°) for *L* = *L*_p_/100 and to 0.44 rad (25°) for *L* = *L*_p_/10. 
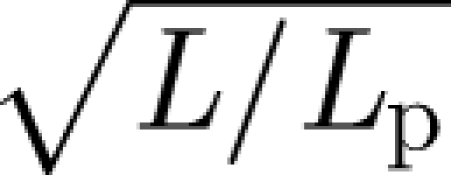
 is a very good approximation of *Δψ* for *L* up to about *L*_p_/3. For yet larger *L/L*_p_ ratios, the thermally induced mean deflection of a filament at its free end is given by arccos[exp(−*L/L*_p_)] (e.g. Howard 2001, p. 111).

### 3.3. Transduction of the torque

From these results, one may conclude that a torque transducer should have a large *mH/kT* ratio in order to reduce the effect of thermal fluctuations on the precision of the measurement and to provide a safety margin for (geological) times during which the intensity *H* of the external field is strongly reduced compared with present-day fields (see fig. 4 in Kirschvink *et al*. 2010). One may be tempted to conclude also that the *mH/K* ratio would ideally be larger than unity, in order to allow for large deformations and to remove the axial ambiguity of the system with respect to the parallel and antiparallel orientation of the external field with respect to the magnet's rest orientation. As shown, the parallel orientation always represents a stable minimum, while the antiparallel orientation of the magnet to the external field changes from a metastable to a labile configuration as the *mH/K* ratio crosses unity. If the system is in the *mH/K* > 1 regime, the 180° orientation of the external field can be detected—and with it not just the axial orientation of the field in space, but also its polarity—by the sudden increase in the fluctuation amplitude of the deflection when the field orientation approaches *θ*_0_ = 180°. Binhi (2006), who focused on the labile state under the *θ*_0_ = 180° orientation (i.e. when *mH/K* ratios are larger than unity), showed that a small angular deviation *η* of a few degrees from that position could easily be detected by comparing the fluctuation amplitudes at *θ*_0_ = 180° and *θ*_0_ = 180° − *η*. It should be noted that Binhi's equations emerge naturally from our thermofluctuation analysis, a fact that may reassure those readers who have a sceptical attitude towards the theory of stochastic resonance, which Binhi (2006) used as framework.

#### 3.3.1. Chemical transduction of magnetic torque

**Figure 3.**
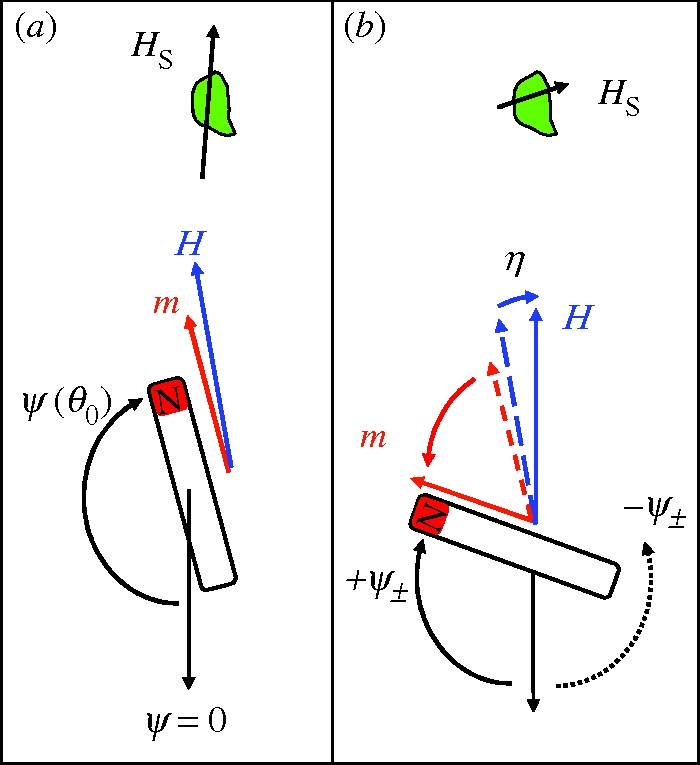
Indirect torque-transducer mechanism according to Binhi (2006). (*a*) Equilibrium deflection *ψ*(180° − *η*) of the magnet for a field orientation *θ*_0_ = 180° − *η* close to the critical orientation *θ*_0_ = 180° (i.e. *η* is a few degrees). (*b*) As *θ*_0_ changes from 180° − *η* to 180°, the magnet suddenly recoils to the +*ψ*_±_ orientation. This way, a small change in external field direction can produce a large change in the magnet's deflection, hence a large change in the magnet's strayfield *H*_s_ at the site (green patch) where free-radical reactions are assumed to transduce the strayfield into a nerve signal. Thermal fluctuations can drive the magnet from its pre-critical *ψ*(180° − *η*) orientation also into the metastable −*ψ*_±_ orientation (dotted arrow). If the energy barriers between the +*ψ*_±_ and the −*ψ*_±_ state are of the order of the thermal energy, the magnet will repeatedly bounce between the +*ψ*_±_ and −*ψ*_±_ state.

As far as the transduction is concerned, Binhi (2006) assumed the rate of intracellular free-radical biochemical reactions to be altered by the strayfield that the intracellular magnet produces in different orientations and concluded that the directional sensitivity achieved that way would be several times better than with the model by Kirschvink (1992), in which the magnet is coupled to a force-gated transmembrane ion channel. It is clear that in order for Binhi's mechanism to work most efficiently, the free-radical reaction sites would have to be distributed very inhomogeneously about the magnet, since the effect of different orientations of the magnet would cancel out for a uniform distribution of reaction sites about the magnet. Let us now assume that we have a cell in which the rest position of the magnet is nearly antiparallel to the external field direction, so that the system is close to its critical state (figure 3*a*). We assume the free-radical reaction sites to be concentrated over a narrow cone extending from the tip of the magnet so that they experience the maximum strayfield intensity for the subcritical field orientation. A rotation by a small angle *η* towards the field axis would bring the system into the labile state *θ*_0_ = 180° (figure 3*b*), from where the magnet's orientation would jump right into the next nearest minimum, which is at **ψ**_*±*_ = *±Δψ*(*θ*_0_ = 180°) (see equations (D 6) and (D 7)). For *ψ*_±_ = 90° (see figure 1*e* and *f*), the field acting on the free-radical reaction sites amounts to just a little more than half the maximum field, according to the dipole formula3.6
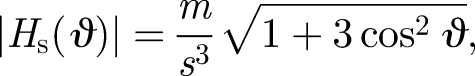

where *ϑ* is the angular distance between the dipole axis and the reaction site, located at a distance *s* away from the centre of the dipole. As long as the radicals are close to the magnet, the contribution of the weak externally applied field to the effective field can be neglected in equation (3.6). The reduction in field strength may shorten the lifetime of radical pairs and reduce the triplet yield (for signal transduction of magnetically induced chemical changes in radical-pair systems, see Weaver *et al*. 2000).

**Figure 4.**
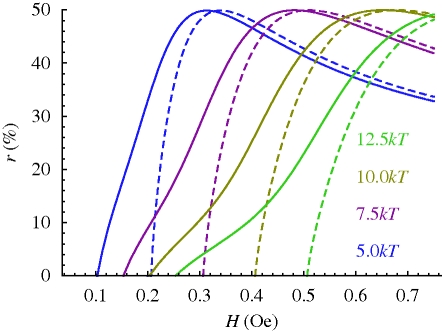
Relative reduction *r*(*H*; *m*, *K*) of strayfield intensity (equation (3.7)) produced by a magnet at a free-radical reaction site on moving from *θ*_0_ = 180° − *η* to 180° (compare figure 3). The magnetic moment of the magnet is fixed (25*kT*/Oe); the values of *K* range from 5 to 12.5*kT*/rad (colours). Dashed lines, *r*(*H*; *m*, *K*) calculated from equations (D 7) and (D 8) (see also Binhi 2006). Solid lines, *r*(*H*; *m*, *K*) calculated from equation (D 9).

It is worth asking if that magnetoreception principle could also be used to detect variations in field intensity. In figure 4, it is shown how the relative reduction of the strayfield intensity at the radical reaction site,3.7
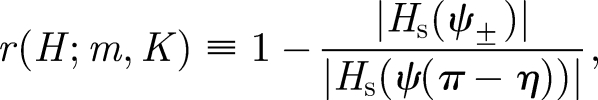

depends on the external field strength *H* for a fixed value of *m* (here 25*kT*/Oe) and various values of *K*. All the *r*(*H*; *m*, *K*) curves have a maximum of 50 per cent at *H* ≅ 1.7*K/m* and asymptotically converge towards 17 per cent when *H* ≫ *K/m* (not shown). Around the point *r*(*H*; *m*, *K*) ≅ 30 per cent (when *H* ≅ *K/m*), the *r*(*H*; *m*, *K*) curves are roughly linear and have their maximum slope. That point therefore has the highest sensitivity to a change in external field intensity and so would define a convenient operation point. The relative sensitivity is obtained as 0.8*δH*/*H*, that is, the absolute sensitivity is better at lower field strength (compare also with equation (3.5)). The measurement of the field intensity *H* with a precision of *δH*/*H* = 10 per cent requires that the free-radical-based transduction pathway has a field sensitivity of 8 per cent.

#### 3.3.2. Mechanosensitive ion channels as transduction elements

As we mentioned earlier, a critical condition for torque transduction through chemoreception is the existence of a labile orientation of the magnet, which in turn requires that the magnetic energy exceed the elastic rigidity of the material to which it is anchored. Also, the rotational motion of the magnet must not be restricted by intracellular components other than the filaments to which it is attached, since the deflection amplitude of the magnet can be expected to be of the order of the length of the magnet. In the following, we show that an effective torque transducer can also be realized in a regime where the magnetic torque is one order of magnitude lower than the elastic spring constants involved. A magnet coupled tightly to the elastic matrix has a fast reaction time to a change in the external magnetic field orientation, primarily because the deflection angle is small in the regime *mH*/*K* ≪ 1 (equation (3.4*a*)). With small deflection angles *ψ*, there is no longer the need for a clear space around the magnet. The minimum space requirement is a cone whose axis coincides with the long axis of the magnet and whose opening angle is 2*ψ*. More importantly, a system in which a magnet coupled to a relatively stiff elastic system experiences smaller thermal fluctuations, since 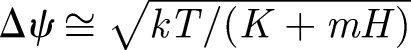 (see equation (D 3*a*)).

**Figure 5.**
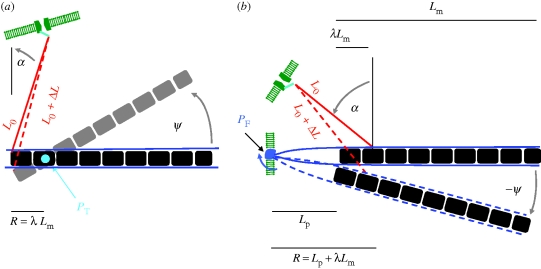
Sketch of mechanosensitive transduction pathway with geometrical parameters used for theoretical modelling (not to scale). A magnetosome chain, which can be deflected about a pivot (stiffness *K*_p_), is connected through a filament to a force-gated ion channel. The orientation *ψ* = 0 defines the orientation of the chain where no elastic torque acts on the pivotal spring. (*a*) The torsional pivot *P*_T_ is oriented perpendicular to the plane defined by the magnet and the gating filament. The pivot is attached to the chain at a distance *R* = *λL*_m_ from the end where the gating filament is inserted. The anchor point *P*_1_ of the gating filament lies on the circle defined by *P*_1_ = −*R*(cos *ψ*, sin *ψ*). The other end of the gating filament is attached to a force-gated ion channel in the cell membrane at position *P*_2_ = (−*R* − *L*_0_ sin *α*, *L*_0_ cos *α*). (*b*) The gating filament is anchored to the magnet at a distance *R* = *L*_p_ + *λL*_m_, where *L*_m_ is the length of the magnet and *λ* is a fraction of *L*_m_. The anchor point *P*_1_ lies on the circle defined by *P*_1_ = *R*(cos *ψ*, sin *ψ*). The other end of the gating filament spring is attached to a force-gated ion channel in the cell membrane at position *P*_2_ = (*R* *−* *L*_0_ sin *α*, *L*_0_ cos *α*). In (*a*,*b*), *ΔL*(*ψ*) is the change in the Euclidian distance between *P*_2_ and *P*_1_ as the chain is deflected by an angle *ψ*. For small angles, *ΔL*(*ψ*) can be approximated as arc length.

In the following, we assume that the magnetic torque is transduced by way of mechanosensitive ion channels. We start out with the proposition by Kirschvink (1992), where the magnet is connected through a filament to a force-gated transmembrane ion channel, so that the magnetic torque acting on the filament is transmitted to the channel (figure 5). Depending on the geometrical and structural constraints, the permanent magnet (e.g. a magnetosome chain) can be directly anchored to the gating spring or mechanically coupled to it through a relatively stiff filament, connected in series. The two elastic elements connected in series experience the same force when the magnet is deflected about the pivot, while the strain in either element is proportional to the spring constant of the other element. Thus, with a stiff connecting filament, almost all the strain will be taken up by the weak element, i.e. the system gate + ion channel. It is clear that in order to convert the magnetic torque into a large force, the lever arm *R* needs to be short, which can be achieved by inserting the connecting filament and the pivot close to one another near one end of the chain. In figure 5*a*, the pivot is a torsional one, which twists upon a deflection of the magnet about the pivot. Importantly, since the pivot axis is normal to the plane defined by the magnet and the filaments, the lever arm can be made arbitrarily small. In figure 5*b*, however, the pivot axis is in the same plane as magnet and filaments, so the lever arm *R* is necessarily longer than it is in figure 5*a*. The deflection of the chain about the pivot in figure 5*b* can bend either the membrane about the insertion point of the pivotal filaments (sketched in blue) or the pivotal filaments themselves. However, in order to achieve a short lever arm, the pivotal filaments need to be short too, which increases their flexural rigidity. It is therefore energetically more favourable to bend the membrane about the pivot point rather than to bend the pivotal filaments. Readers familiar with hair cells will note a superficial resemblance of the structure shown in figure 3*b* with a stereociliary pivot, yet there is an important difference. The stereociliary pivot of hair cells consists of a number of short actin filaments anchored firmly in the cuticular plate of the hair cell. That construction gives rise to a flexural stiffness of 0.5 × 10^−15^ N m rad^−1^ for an individual stereociliary pivot, as determined on hair cells of the bullfrog's sacculus (Howard & Ashmore 1986). Similar values were obtained on hair cells of the cochlear duct of cooter turtles (Crawford & Fettiplace 1985). To put into perspective the stiffness value quoted, we express it in terms of the thermal energy (*kT* = 4.3 pN nm), which yields about 10^5^*kT/*rad. This consideration vitiates the surprising result obtained by Edmonds (1992), who concluded that a single hair cell loaded with a single-domain magnetite crystal (*m* ∼ 1 *kT*/Oe) would be enough to precisely detect fluctuations of the geomagnetic field strength of the order of 1 per cent. Edmonds (1992) obtained this figure from balancing magnetic and gravitational couples, without taking into account elastic torques (e.g. due to bending). We argue that the result of such a torque balance is not the field sensitivity, but the field strength below which gravitational force couples become comparable in magnitude to magnetically produced couples (see also appendix E). Nonetheless, Edmonds' (1992) null-detector principle is not affected by that consideration. To avoid misunderstanding, we emphasize that our model—although it has mechanosensitive structures analogous to transduction units in hair bundles—is not a magnetite-loaded hair bundle in which magnetoreception would be a useful ‘side effect’ of acceleration measurements in the inner ear. Instead, we postulate that a magnetite-based sensory cell serves the specific purpose of magnetoreception. In this context, it is also interesting to note that despite extensive ultrastructural work on ciliary bundles of hair cells (e.g. Kachar *et al*. 2000), magnetite crystals have not been found to be associated with cilia.

To quantitatively assess the viability of the models depicted in figure 5, we need to include the energy required to change the open probability *p*^o^ of a force-gated transmembrane ion channel. Typically, the mechanical work required to change *p*^o^ from 50 to 70 per cent is of the order of 1*kT*, which corresponds to a force of 1 pN acting over a distance of 4 nm (Corey & Howard 1994). A force of 1 pN can easily be produced with a magnetosome chain provided that the lever arm is short. If, for example, the magnetic moment of the chain is 25*kT*/Oe, an effective lever arm *R* cos *α* of 50 nm produces 1 pN when a field of intensity *H* = 0.5 Oe is applied perpendicular to the chain. Of course, by reciprocity (actio = reactio), the magnetosome chain has to withstand that force which might be expected to deflect the first crystal of the chain out of the chain axis (figure 5*b*). However, as shown in Shcherbakov *et al*. (1997), crystals in a magnetosome chain are strongly coupled to each other by magnetostatic interactions. Provided that the gap size is small between adjacent magnetosomes, the attraction force is 2*πM*_s_^2^*a*^2^, where *M*_s_ is the saturation magnetization (480 G for magnetite) and *a*^2^ is the cross-section area. For *a* = 50 nm, the attraction force is of the order of 200 pN. Further, we assume the chain to be elastically supported by filaments (as in magnetic bacteria, see Kobayashi *et al*. 2006; Scheffel *et al*. 2006); so we need not be concerned about kinks in the magnetosome chain. As shown in appendix E, gravitational torques can be neglected in the torque balance.

Following the classical model by Howard & Hudspeth (1988), we write the open probability of the ion channel connected to the filament (figure 5) as3.8
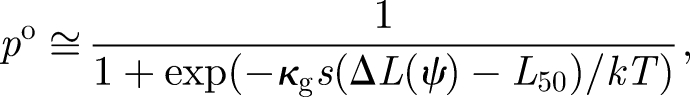

where *κ*_g_ is the stiffness of the gating spring (0.5 pN nm^−1^; e.g. Howard & Hudspeth 1988), *s* is the swing of the gating spring, i.e. the distance by which the gating spring moves between open and closed channel (*s* = 4 nm; Howard & Hudspeth 1988), *ΔL*(*ψ*) is the displacement of the filament from its rest position and *L*_50_ is the midpoint of the opening transition, which may change because of adaptation. Equation (3.8) may contain an additional term to account for changes in the chemical potential of the channel between its open and closed state, but that is usually neglected. For small angles *ψ*, we see from figure 4 that *ΔL*(*ψ*) can be approximated by the arc length *Rψ* projected on *L*_0_, so that the related force is obtained3.9
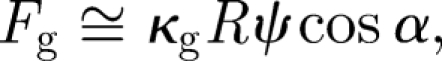

which in turn produces a torque *D*_g_ ≅ *F*_g_*R* cos *α* about the pivot. Hence, we can use3.10
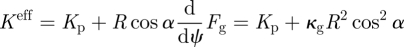

as effective rigidity when seeking the mechanical equilibrium value of *ψ* according to equation (3.4*a*) or (3.4*b*). *K*_p_ in equation (3.10) is the rigidity related to the pivot. When *R* cos *α* = 50 nm, the *κ*_g_*R*^2^ cos^2^ *α* term in equation (3.10) with *κ*_g_ = 0.12*kT*/nm^2^ contributes about 300*kT*/rad to the effective stiffness, in which case *K*^eff^ will be dominated by *κ*_g_*R*^2^ cos^2^ *α* as long as the pivotal stiffness *K*_p_ is less than about 100*kT*/rad.

**Figure 6.**
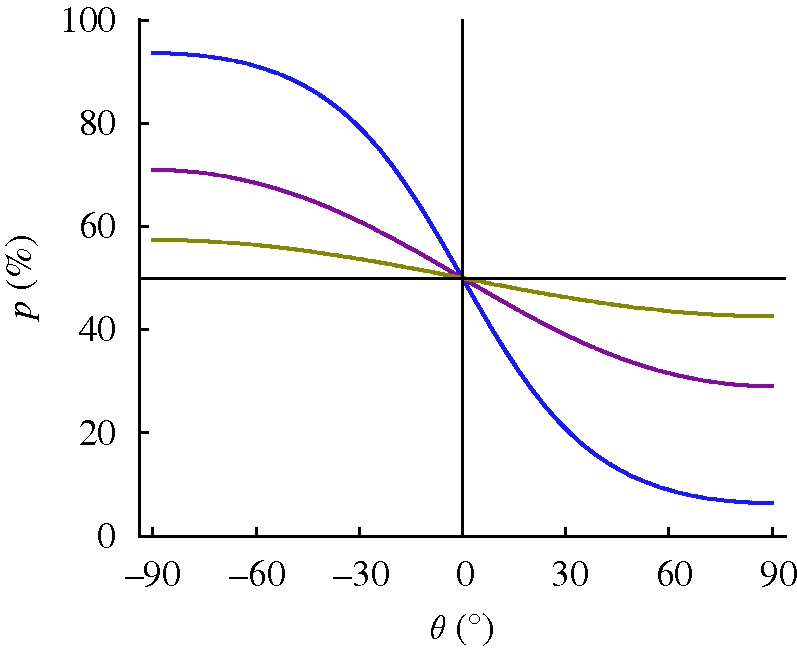
Opening probability of a force-gated ion channel as a function of the orientation of the external field for three different values of the magnetic to thermal energy ratio (olive, *mH* = 5*kT*; magneta, *mH* = 15*kT*; blue, *mH* = 45*kT*). Parameters: stiffness *K*_p_ of the pivot 100*kT*/rad, effective lever arm and *R* cos *α* = 50 nm, i.e. *K*^eff^ = 390*kT*/rad.

From figure 6, it can be seen that the opening probability of a force-gated ion channel changes from 50 to 70 per cent on varying the field orientation from 0° to 90° for *mH* = 15*kT*, *K*_p_ = 100*kT*/rad, *R* cos *α* = 50 nm and *K*^eff^ = 390*kT*/rad. The key point here is that the ratio of the magnetic torque *mH* to the effective elastic stiffness *K*^eff^ amounts to only 4/100, and yet, the magnetically produced force of 1.0 pN is sufficient to significantly alter the opening probability of the channel. That this mechanism is efficient can be seen by comparison with the mechanism modelled in Solov'yov & Greiner (2007), which produces a force of only a few tenths pN upon a 90° rotation of the magnetic field, although the magnetic energy contained in the modelled chain of platelets was as large as 580*kT*. Solov'yov & Greiner (2007) assumed a chain of 10 particles, each of dimensions 1 × 1 × 0.1 µm^3^ and of fixed magnetization intensity of 50 G, and computed the chain's strayfield and its attraction force on a nearby cluster of superparamagnetic magnetite crystals. We argue that if the chain of platelets has the properties as assumed by Solov'yov & Greiner (2007), the torque on the chain produced by a 90° shift of the external field will be a first-order effect that makes the indirect strayfield mechanism an effect of second order in magnitude. We refer to Shcherbakov & Winklhofer for an alternative model on the basis of a chain of platelets.

#### 3.3.3. Membrane-stress-activated ion channels as transduction elements

The second kind of mechanosensitive ion channels are those that open (or close) in response to stress received from the lipid bilayer membrane in which they are embedded. These are found in all animals and all sorts of cells and are associated with intrinsic cell transduction (Markin & Sachs 2004). Because of their ubiquity, those channels have been proposed to act as transducers of mechanical stimuli produced by magnetic torques (Walker *et al*. 2002) or forces (Davila *et al*. 2003). According to the classification scheme of mechanosensitive ion channels by Markin & Sachs (2004), there are three end member types: (i) area-sensitive channels, activated by tensile stress perpendicular to the ion channel axis, (ii) shape-sensitive channels, activated by a torque in the membrane (i.e. due to bending), and (iii) length-sensitive channels, activated by a line tension along the channel axis. As Markin & Sachs (2004) point out, natural mechanosensitive channels may combine two or even three of these basic deformation types, and it depends on the generalized force (tension, torque or line tension) as to which deformation mode is activated. Let us now consider the application of a single point force to the cell membrane, transmitted through the insertion point of a thin filament that is attached at its other end to the magnetosome chain. The cell membrane can be approximated by a spheroidal shell, and it is known from the theory of shells that an elastic shell cannot be bent without being stretched (e.g. Landau & Lifshitz 1991, ch. 15). Let the characteristic deflection amplitude produced by the point force *F* be *ξ* ∼ *F*/*k*_m_, where *k*_m_ is the membrane spring constant, with *k*_m_ ∼ *Eh*^2^*/R*_c_, where *E* is the Young modulus, *h* is the thickness of the membrane and *R*_c_ is the radius of curvature of the membrane when no force is applied. The deflection *ξ* produces a tension ∼ *ξ*/*R*_c_ and a local curvature of ∼ *ξ*/*hR*_c_. Although the stretch energy ∼ *Eh*(*ξ*/*R*_c_)^2^ and bending energy ∼ *Eh*^3^(*ξ*/*hR*_c_)^2^ are of the same order, the amount of stretching is much smaller than the amount of bending! Thus, the application of a point force pays mostly in bending deformation and therefore is more likely to activate shape-sensitive rather than area-sensitive ion channels. Of course, if an area-sensitive ion channel is located at the right spot (where the stretch energy is concentrated), it can be opened as well. For a lipid bilayer membrane supported by a soft cytoskeleton network, the spring constant *k*_m_ was estimated to be approximately 0.1 pN nm^−1^ (Boulbitch 1998). This is comparable with the stiffness *κ*_g_ of a gating spring. Thus, if the force *mH* sin *θ*/(*R* cos *α*) emerging at the short lever arm *R* of a magnetosome chain is sufficient to open a filament-gated channel, it is also sufficient to locally bend the membrane. With *k*_m_ ∼ 0.1 pN nm^−1^, a force of 1 pN magnitude is sufficient to produce a membrane deflection of the order of the membrane thickness and thus to induce membrane buckling—as indicated by the change in local curvature (1−*ξ*/*h*)*R*_c_ when *ξ*/*h* >1. The buckling transition could define a suitable set point for a magnetoreceptor.

Finally, we note that the pivot itself may be the active element that mediates the membrane tension, in which case no ‘connecting filament’ (red rod in figure 5*b*) is needed. Deflection of the magnet produces a force couple around the pivot's insertion point in the membrane, which again can alter the opening probability of mechanosensitive ion channels in that membrane patch.

## 4. Conclusions

The torque mechanisms on the basis of single-domain magnetite analysed in §3.3 can be expected to produce physiologically exploitable signals, for a large range of magnetic to elastic energy ratios. To chemically transduce the strayfield variations produced by a deflected intracellular magnet (Binhi 2006), it is necessary that the magnet be coupled to a soft elastic matrix that allows for large deflections in the first place. In contrast, if the torque mechanism is directly coupled to a mechanotransductive pathway, the rigidity of the mechanosensitive structures (equation (3.10)) is an essential part of the torque balance and will typically be of the order of 10^2^*kT*/rad per connecting filament. As a consequence, the resulting angular deflections of the magnet will be small. As shown in §3.3.2, however, the decisive parameter is not the angular deflection, but the force produced by the magnetic torque at the short lever arm, or the force couple produced at the insertion point of the pivot. As long as the rigidity of the pivot is smaller than the rigidity of the mechanosensitive structures, the mechanotransductive pathway through a connecting filament provides an efficient way of transmitting the magnetic torque to the mechanosensitive structures.

Whether the magnet is softly or rigidly anchored (or not anchored at all) can be tested on isolated candidate receptor cells in experiments similar to those conducted on magnetic bacteria with rotating magnetic fields under the light microscope (e.g. Hanzlik *et al*. 2002). A cell is first aligned with a sufficiently strong field, say 10 Oe, from which the orientation time can be determined. Then the field is slowly rotated in the optical plane. Since the cell has a much larger viscous resistance factor than the magnet inside the cell, the magnet would—if only softly anchored—rotate into its equilibrium position before the cell responds to the field change by passive rotation and one can expect to see a qualitatively different orientation behaviour for such a cell than for a cell in which the magnet is more rigidly anchored. If the magnet is not anchored at all, it cannot transmit a torque to the cell membrane and thus the application of a rotating homogeneous field will not rotate the cell.

Although behavioural experiments using pulsed magnetic fields are immensely useful to demonstrate the involvement of magnetic material in the orientation behaviour (see §2.3), they have limited diagnostic power to resolve whether the magnet is softly or rigidly anchored. All torque mechanisms presented here would be affected by a magnetic pulse, but—provided that no damage has occurred—it depends on the kind of signal produced by the mechanism whether the effect is only immediate or transient, i.e. it depends on whether the mechanism is sensitive to the polarity of the torque vector or just to the magnitude of the torque (§§2.2 and 2.3).

Curiously, a magnetic torque receptor coupled to a chemical transduction pathway (as in Binhi's model) has the potential to act as a polar compass, even though the radical-pair reactions *per se* cannot be harnessed to resolve the polarity. Although this conclusion may appear counterintuitive, it becomes clear when considering that it is not the axial sensitivity of radical-pair reactions which is exploited in Binhi's model, but the sensitivity of radical-pair lifetime and reactivity to the field strength.

Whether a torque receptor coupled to a mechanosensitive channel has polar or axial characteristics depends on whether or not the mechanosensitive transduction pathway has a polarity. If the mechanosensitive channel is directly gated through a filament (as shown in figure 5), the open probability depends on the direction of the magnetically produced force that acts on the gating filament and therefore depends on the polarity of the external field. If, however, the ion channels are not directly gated by a filament but indirectly by way of membrane deformations produced by a filament inserted nearby, it depends on the local stress field that acts on the channel. If the ion channel is area-sensitive (stretch activated), it is likely to feel the same stress independent of the direction of the force transmitted to the membrane; hence, we expect an axial response of a magnetoreceptor coupled to stretch-activated channels. Thus, an inclination compass can be realized on the basis of a torque detector coupled to a stretch-sensitive ion channel that measures the magnitude of the torque. For a hybrid torque detector, which contains both superparamagnetic and permanent magnetic material, the situation is more complicated because the axis of the susceptibility tensor may not coincide with the axis of the permanent magnetic moment. For example, the hybrid receptor proposed by Solov'yov & Greiner (2007) was found to have features of the inclination compass at certain conditions, but not generally (Solov'yov & Greiner 2009). When it comes to extracting the field direction from the mechanically transduced torque, it is also important to remember that the instantaneous torque −*mH* sin *θ*_0_ is a non-unique function. Even if magnetic moment and field strength are known, a virtual field applied at the supplementary angle 180° − *θ*_0_ would produce the same torque as the original field applied at *θ*_0_. The twofold ambiguity also applies to the equilibrium deformation as long as the instantaneous magnetic torque is smaller than the counteracting elastic rigidity. If the torque detector is used as a null detector—as suggested by Edmonds (1992)—the 0° and the 180° field orientation are equivalent, so that all torque detectors discussed here except the one suggested by Binhi (2006) would measure the axial orientation but not the polarity of the field. Note that the ambiguity does not exist for the *θ*_0_ = 90° field orientation (maximum torque position), for which ‘polar’ or ‘axial’ again depends on whether the full torque vector or its magnitude is transduced.

The torque-transducer models analysed in §3.3 are also sensitive to intensity variations of ambient magnetic field (figures 4 and 6), although the output signal may be non-nonlinear (figure 6) or even non-monotonic (figure 4), so that functionality of a given receptor cell may be limited to certain intensity window. Although any magnetic torque detector will be subject to thermal fluctuations, these are not debilitating to the magnetoreception mechanism. If the torque receptor is coupled to a mechanosensitive ion channel, the transduced signal will certainly fluctuate, but importantly, it will fluctuate about a stable average value. This is the fundamental difference to a system that is not subject to an orienting force. The thermal fluctuation amplitude can be used as an inverse measure of the field intensity (see expressions (D 3*a*) and (D 3*b*)). In Binhi's (2006) model, thermal fluctuations even help to increase the sensitivity of the system.

## Appendix A. Apparent and intrinsic susceptibility

The apparent magnetic susceptibility *χ*′ is defined as the slope of the magnetization curve, *χ* = (d*M/*d*H*). It depends on the geometry of the magnetic body and is related to the intrinsic magnetic susceptibility *χ* of the magnetic material in the body by the following expression:

A 1

where *N*_*i*_ (*i* = 1, 2, 3) is the demagnetization factor along the *i*th principal axis of the body. If the magnetic volume concentration of the body is *c*, then expression (A 1) takes the form

A 2

We approximate the magnetic layer of the otolithic membrane as a flat ellipsoid (*a* > *b* ≫ *c*) with aspect ratios *b/a* = 1.25/2.5 and *c/a* = 0.05/2.5. Using the formulae for the demagnetization factors of the general ellipsoid, given by Osborn (1945), we obtain *N*_a_/4*π* = 0.0122, *N*_b_/4*π* = 0.0343 and *N*_c_/4*π* = (1 − *N*_b_/4*π* − *N*_a_/4*π*) = 0.953. The magnetic torque **D**_*χ*_ = *V*(*χ*′·**H**) × **H** is given by

A 3

where *θ* is the colatitude and *ϕ* is the azimuth of **H** with respect to the {**e**_*a*_, **e**_*b*_, **e**_*c*_} coordinate system, i.e. *θ* is the angle between **H** and **e**_*c*_, and *ϕ* is the angle of the projection of **H** onto the (**e**_*a*_, **e**_*b*_) plane, reckoned from **e**_*a*_. With *χ*′_*a*_ > *χ*′_*b*_ ≫ *χ*′_*c*_, the maximum out-of-plane couple is produced by *D*_*b*_ and occurs when the field is oriented at (*ϕ* = 0°, *θ* = ±45°) or (*ϕ* = ±180°, *θ* = ±45°), i.e. 1/2(*χ*_*c*_′ − *χ*_*a*_′)*H*^2^*V*. The maximum value of the in-plane couple, produced by *D*_*c*_, occurs at (*ϕ* = ±45°, *θ* = ±90°) or (*ϕ* = ±135°,*θ* = ±90°) and is given by 1/2(*χ*_*a*_′ − *χ*_*b*_′)*H*^2^*V*.

## Appendix B. Torque characteristics

If the torque transducer is based on magnetic material that can be reversibly magnetized (i.e. it has no magnetic remanence), the magnetic torque is given by equation (2.2) or, more specifically, by equation (A 3). The *H*^2^ dependence of the torque in equation (A 3) suggests that the polarity of **H** does not matter here. Indeed, we see that

B 1a

i.e. **D**_*χ*_ is invariant to a full reversal of the external magnetic field. Further,

B 1b

which expresses that the torque produced in a field with inverted horizontal field component is the same as the torque produced in a field with inverted vertical component and that both situations produce a torque different to the torque produced under normal field conditions. If either component is inverted with respect to the normal field, then the direction of the torque rotates horizontally by 180°,

B 2

where **R**(−*π*) is the diagonal matrix

which obviously represents a rotation of the coordinate system by 180° about the vertical axis. The set of relations (B 2*a*,*b*) is in *exact* agreement with the orientation behaviour of migratory birds reported in the classic paper by Wiltschko & Wiltschko (1972).

If the torque transducer is based on permanent magnetic material, then the torque is given by

B 3

where **m** = (*m*_*x*_, *m*_*y*_, *m*_*z*_) is the magnetic moment, **H** is the external magnetic field **H** = (cos *I* cos *D*, cos *I* sin *D*, sin *I*)*H*, where *D* is the declination and *I* is the inclination. Equation (B 3) depends only linearly on *H*, so that the equivalence relations (B 1*a*,*b*), which hold for **D**_*χ*_, now apply to the magnitude of **D**_m_, but not to **D**_m_ itself:

B 4a

B 4b

The same applies to the projection of the force couple onto a filament that connects the magnet to a mechanosensitive structure. Let us now define the two vectors **a** = (***e***_m_ × **m** × **H**)/*R* and **n**, where **a** is the force exerted by the magnetic torque onto a filament attached at a distance *R* from the pivot and **n** is the orientation vector of the filament. Again, the projection **a**·**n** does not satisfy the equivalence conditions, whereas its magnitude |**a**·**n**| does.

## Appendix C. Thermodynamic equilibrium deflection

The energy function has the generic form

C 1

where *K* is the effective rigidity of all elastic elements connected to a permanent magnet of magnetic moment *m* in a field *H*. The thermodynamic equilibrium value of the observable *ψ* is the expectation value of *ψ*, where each possible deflection *ψ* is weighted according to its Boltzmann probability

C 2

where the normalizing factor *Z* is the partition sum

C 3

The integral extends over all states, although it is usually sufficient to set [−2*π*; 2*π*] as integration limit, since *ψ* values outside that range soon become energetically so unfavourable that their Boltzmann probability and thus their weight are negligible. Another advantage of the thermodynamic treatment is that it furnishes also the magnitude of the fluctuations about the equilibrium value

C 4

where *Δ**ψ* is the standard deviation of the fluctuations about the mean value 〈*ψ*〉 and 〈*ψ*^2^〉 is the mean-square value of *ψ*, given by

C 5

## Appendix D. Thermofluctuation analysis

We start from the expression for the total energy (equation (C 1)). For the two field orientations *θ*_0_ = 0 (always stable) and *θ*_0_ = *π* (metastable or labile, depending on *mH/K*), the potential *W*(*ψ*) is reflection symmetric about the equilibrium deflection *ψ* = 0, which allows us to expand *W*(*ψ*) into a series of even powers of *ψ* about *ψ* = 0, i.e.

D 1a

D 1b

From the different coefficients of the *ψ*^2^ term, we see that elastic stiffness *K* is apparently increased (equation (D 1*a*)) or decreased (equation (D 1*b*)) by *mH*. The energy well about the deflection *ψ* = 0 is indeed shallower for the antiparallel orientation (metastable state) than for the parallel orientation (stable state), and therefore thermal perturbations cause greater angular scatter in the antiparallel orientation. Once the *mH/K* ratio exceeds unity, the metastable state turns into a local maximum, which implies a transition from a one-well to a double-well system.

An estimate of the mean-square angular deviation of *ψ* may be obtained by determining the value of *ψ*^2^ that changes the total energy according to equations (D 1*a*,*b*) by *kT*/2, which corresponds to the average thermal energy for one degree of freedom. For the stable orientation *θ*_0_ = 0, we can neglect all terms in equation (D 1*a*) that are of order *ψ*^4^ or higher and have

D 2

which we could have directly obtained by applying the equipartition principle to a spring with effective spring constant (*K* + *mH*). Owing to the symmetry of the potential, the mean value 〈*ψ*〉 is zero (both for *θ*_0_ = 0 and *θ*_0_ = *π*) so that the angular variance (*Δ**ψ*)^2^ is identical to 〈*ψ*^2^〉 (equation (C 4)). Thus, the thermally induced angular scatter about the stable *θ*_0_ = 0 orientation is given by

D 3a

To approximate the fluctuations in the antiparallel orientation, we have to take into account also the *ψ*^4^ term in equation (D 1*b*), because *Δ**ψ*(*θ*_0_ = *π*) would diverge in the limit *mH* → *K* if we used an effective spring constant of (*K* − *mH*). Applying the equipartition principle to expression (D 1*b*), we have

where each series coefficient is weighted by the order of *ψ*. Thus, the mean-square angular deviation about the *θ*_0_ = *π* orientation is

D 3b

which is valid for both *mH/K* < 1 (metastable) and *mH/K* > 1 (labile). Up to about *mH/K*_p_ ≤ 0.5, the ratio of the angular scatter in the antiparallel to the parallel state may be approximated as

D 4

In the limit *mH* → *K*, we have *Δ**ψ* (*θ*_0_ = *π*) = (6*kT*/*mH*)^1/4^ and thus

D 5

We have determined that ratio on the basis of equation (C 5) also using the untruncated expression for the total energy (C 1) and obtained the same functional dependence as in equation (D 5). That procedure yields a numerical prefactor of 1.83, which is smaller by a factor of 0.8 than the one in expression (D5). This small discrepancy is attributable to equation (D 3*b*), which slightly overestimates the actual angular variation, while equation (D 3*a*) is accurate as long as *mH* = *K* ≥ *kT*. For *mH* = *K* ≪ *kT*, expression (D 3*a*) diverges, while the partition-sum-based estimate converges towards the value 3.63. Clearly, that parameter range is certainly not of interest here. It is also interesting to consider the non-thermal (*kT* = 0) limit of equation (D 3*b*), i.e. the macroscopic situation, where we simply have

D 6

for *mH/K* > 1, while *Δ**ψ*(*π*) = 0 for *mH/K* ≤ 1. In the non-thermal limit, *Δ**ψ*(*θ*_0_ = *π*) is the angular distance between the stable *ψ* = 0 position and the position *ψ*_±_ at which the energy is minimum (i.e. in either well of the double-well potential). Interestingly, Binhi (2006), who treated the double-well situation, derived the expression

D 7

to provide

D 8

as an estimate of the minimally detectable angle *η* from the antiparallel position *θ*_0_ = *π*. However, that expression diverges as soon as *mH* becomes equal to or less than *K*, i.e. when the energy landscape switches from the double-well to the single-well shape, for which Binhi's formulae do not apply. For *mH* ∼ *K*, we suggest using the following expression for the minimally detectable angle:

D 9

From equations (3.6) and (3.7) in the article, we see that the value of *η* for which the thermofluctuation mechanism becomes inefficient is given by the condition cos^2^*Δ**ψ*(*π*) = cos^2^*ψ*(*π* − *η*), which is the case when *η* becomes as large as the change in deflection when going from *θ*_0_ = *π*− *η* to *θ*_0_ = *π*.

## Appendix E. Role of gravity

The gravitational couple acting on the pivot owing to the permanent magnet (volume *V* and length *L*_m_) is *D*_g_ = (1 − *λ*)*L*_m_(*ρ*_m_ − *ρ*_c_)*gV* cos *β*, where (1 − *λ*)*L*_m_ is the lever arm, *ρ*_m_ − *ρ*_c_ is the density difference between the magnetic material (magnetite, *ρ*_m_ = 5 g cm^−3^) and the cytosol (approx. 1 g cm^−3^), *g* is the gravitational acceleration (approx. 980 cm s^−2^) and *β* is the angle between the magnet and the vertical. Let us assume that the magnet is a single-stranded chain of *N* = 50 tightly spaced single-domain magnetite particles, each of dimensions 60 × 50 × 50 nm^3^. This gives a total length *L*_m_ = 3.5 µm and a magnetic-to-thermal energy ratio of *mH* = 50*kT* at *H* = 0.5 Oe. The maximum gravitational couple is smaller than *kT*/3 and thus negligible, unless the field strength is lower than 0.01 Oe. The situation is different in the dendrite containing clusters of superparamagnetic magnetite, which extend over tens of micrometres. A detailed analysis of the interplay between magnetic and gravitational couples in such a macroscopic system was provided in Shcherbakov & Winklhofer (2004).
